# TBI+rATG为基础的联合预处理方案单倍体造血干细胞移植治疗化疗耐药进展期外周T细胞淋巴瘤11例

**DOI:** 10.3760/cma.j.issn.0253-2727.2023.07.010

**Published:** 2023-07

**Authors:** 榕 徐, 肃 李, 慧霞 刘, 道林 魏, 瑛 蒋, 京京 王, 素素 刘, 椿 王, 骏 朱

**Affiliations:** 1 上海力泉医院血液科，上海 201418 Hematology Department of Shanghai Liquan Hospital, Shanghai 201418, China; 2 上海闸新中西医结合医院血液科，上海 200040 Hematology Department of Shanghai Zhaxin Integrated Traditional Chinese and Western Medicine Hospital, Shanghai 200040, China

**Keywords:** 外周T细胞淋巴瘤, 单倍体造血干细胞移植, 预处理, 全身放射治疗, 抗胸腺细胞球蛋白, Peripheral T-cell lymphoma, Haplo-HSCT, Conditioning, Total body irradiation（TBI）, Anti-thymocyte globulin

## Abstract

**目的:**

评估以全身放射治疗（TBI）+兔抗人胸腺细胞免疫球蛋白（rATG）为基础预处理方案的单倍体造血干细胞移植（haplo-HSCT）治疗化疗耐药进展期外周T细胞淋巴瘤（PTCL）的疗效与安全性。

**方法:**

纳入2019年9月至2022年12月在上海力泉医院和上海闸新中西医结合医院血液科接受TBI+rATG为基础预处理方案haplo-HSCT的11例化疗耐药进展期PTCL患者，对其临床资料进行回顾性分析。

**结果:**

①11例患者中男6例，女5例，中位年龄40（22～58）岁；外周T细胞淋巴瘤（非特指型）6例，血管免疫母细胞性T细胞淋巴瘤3例，蕈样真菌病（大细胞转化）1例，T大颗粒淋巴细胞白血病1例；Lugano分期均为Ⅲ、Ⅳ期；8例患者有B症状；移植前中位化疗线程数为4（2～10）线，移植前疾病状态均为疾病进展（PD）。诊断至移植的中位时间为17（6～36）个月。②预处理方案：TBI总量10 Gy；rATG 2.5 mg·kg^−1^·d^−1^、−5 d～−2 d；依托泊苷15 mg·kg^−1^·d^−1^，−5 d、−4 d，环磷酰胺50 mg·kg^−1^·d^−1^，−3 d、−2 d。原发病中枢神经系统侵犯的患者，将依托泊苷和环磷酰胺替换为塞替派（5 mg·kg^−1^·d^−1^，−5 d、−4 d）并加用阿糖胞苷（2.0 g/m^2^每日2次，−3 d、−2 d）。③所有患者均成功植入，中位中性粒细胞植入时间为14.5（11～16）d，中位血小板植入时间为13（8～18）d。1例患者发生Ⅰ～Ⅱ度急性移植物抗宿主病（GVHD），1例患者发生Ⅲ/Ⅳ度急性GVHD，8例存活患者中4例发生慢性GVHD。④移植后有9例患者获得完全缓解（CR）。⑤所有患者预处理后发生造血抑制，3例出现腹泻，4例发生黏膜炎，3例转氨酶/胆红素升高，7例发生感染，经过治疗均得到缓解。⑥移植后1年非复发死亡率（NRM）为（22.5±14.0）％，累积复发率（CIR）为（20.2±12.7）％，总生存（OS）率为（72.7±13.4）％，无病生存（DFS）率为（63.6±14.5）％。

**结论:**

TBI+rATG为基础预处理haplo-HSCT是化疗耐药进展期PTCL有效且安全的治疗方法。

## Abstract (en)

**Objective:**

To evaluate the clinical outcomes and safety of haploidentical hematopoietic stem cell transplantation（haplo-HSCT）using a conditioning regimen based on total body irradiation（TBI）and rabbit anti-human thymocyte globulin（rATG）in the management of chemotherapy-resistant advanced peripheral T-cell lymphoma（PTCL）.

**Methods:**

Clinical data of 11 patients with chemotherapy-resistant advanced PTCL who underwent haplo-HSCT with a TBI+rATG-based conditioning regimen at the Department of Hematology, Shanghai Liquan Hospital and Shanghai Zhaxin Integrated Traditional Chinese and Western Medicine Hospital, from September 2019 to December 2022 were retrospectively analyzed.

**Results:**

①Among the 11 patients（six males and five females）, with a median age of 40 years（range: 22–58 years）, there were six cases of PTCL, not otherwise specified（PTCL-NOS）, three cases of angioimmunoblastic T-cell lymphoma（AITL）, one case of large-cell transformation of mycosis fungoides（MF-LCT）, and one case of T-cell large granular lymphocytic leukemia（T-LGLL）. According to the Lugano staging system, all patients were in stage Ⅲ or Ⅳ, and eight patients had B symptoms. Before transplantation, the median number of prior lines of chemotherapy was 4（range: 2–10）, and all patients had progressive disease（PD）. The median time from diagnosis to transplantation was 17 months（range: 6–36 months）. ②The conditioning regimen consisted of a TBI dose of 10 Gy, administered at 2 Gy on day −8 and 4 Gy from day −7 to day −6, rATG was administered at a daily dose of 2.5 mg/kg from day −5 to day −2. Etoposide（VP-16）was given at a dose of 15 mg/kg/d from day −5 to day −4, while cyclophosphamide（CTX）was administered at a dose of 50 mg/kg/d from day −3 to day −2. In patients with central nervous system involvement, etoposide and cyclophosphamide were replaced with thiotepa（TT）at a dose of 5 mg/kg/d from day −5 to day −4. Additionally, cytarabine（Ara-C）was added at a dose of 2.0 g/m^2^ twice a day from day −3 to day −2 into the conditioning. ③Successful engraftment was achieved in all patients, with a median time to neutrophil engraftment of 14.5 d（range: 11–16 d）and a median time to platelet engraftment of 13 days（range: 8–18 days）. Acute graft-versus-host disease（aGVHD）occurred in one patient（grade Ⅰ–Ⅱ）, and another patient experienced grade Ⅲ–Ⅳ aGVHD. Among the eight survivors, four developed chronic GVHD（cGVHD）. ④Post-transplantation, nine patients achieved complete response（CR）. ⑤Hematopoietic suppression occurred in all patients after conditioning, with three experiencing diarrhea, four developing mucositis, three exhibiting elevated transaminase/bilirubin levels, and seven developing infectious complications. These non-hematologic adverse events were effectively managed. ⑥At one year post-transplantation, the non-relapse mortality（NRM）was（22.5±14.0）％, the cumulative incidence of relapse（CIR）was（20.2±12.7）％, and overall survival（OS）rate was（72.7±13.4）％, and disease-free survival（DFS）rate was（63.6±14.5）％.

**Conclusion:**

TBI+rATG-based conditioning regimen for haplo-HSCT is an effective and safe treatment approach for patients with chemotherapy-resistant advanced PTCL.

外周T细胞淋巴瘤（PTCL）是一组高度异质性、源于成熟T细胞的恶性增殖性疾病，除ALK^+^间变大细胞淋巴瘤外大部分亚型预后均较差[1]。CHOP或CHOP样化疗方案目前仍是初治PTCL的标准治疗，但其长期无病生存（DFS）和总生存（OS）均不理想，患者单纯接受化疗治疗5年OS率一般不超过40％，对难治/复发患者挽救性化疗的5年OS率只有10％[2]–[4]。异基因造血干细胞移植（allo-HSCT）可改善部分对挽救性化疗尚有反应、能达到完全缓解（CR）或部分缓解（PR）的难治复发患者的预后[5]。但对于挽救性化疗耐药的进展期PTCL，常规清髓性化疗预处理allo-HSCT疗效很差，移植后4年无进展生存（PFS）率仅为18％[6]。我们应用全身放射治疗（TBI）+兔抗人胸腺细胞球蛋白（rATG）为基础的联合预处理方案单倍体造血干细胞移植（haplo-HSCT）治疗11例化疗耐药进展期PTCL患者，获得良好疗效。

## 病例及方法

1. 病例：本项回顾性研究连续纳入2019年9月至2022年3月上海力泉医院和上海闸新中西医结合医院血液科接受haplo-HSCT的11例化疗耐药进展期PTCL患者。纳入标准：①经过淋巴结或病变组织病理检查确诊为PTCL（2016 WHO标准）。②原发病难治或复发且挽救性化疗无效，仍然处于进展期。③年龄18～70岁。

2. 预处理方案：所有患者均以分次TBI+增大剂量rATG为基础：分次TBI（总量10 Gy）：−8 d予以2 Gy 1次，−7 d、−6 d 予以2 Gy每日2次，剂量率为7.5 cGy/min；rATG（总量10 mg/kg）2.5 mg·kg^−1^·d^−1^，−5 d～−2 d静脉滴注。无原发病中枢神经侵犯的患者，TBI+rATG基础上联用依托泊苷（Vp16，总量30 mg/kg）15 mg·kg^−1^·d^−1^，−5 d、−4 d静脉泵入和环磷酰胺（CTX，总量100 mg/kg）50 mg·kg^−1^·d^−1^，−3 d、−2 d静脉滴注；伴原发病中枢神经系统侵犯的患者，联用塞替派（总量10 mg/kg，5 mg·kg^−1^·d^−1^，−5 d、−4 d）和阿糖胞苷（Ara-C，总量8.0 g/m^2^，2.0 g/m^2^每日2次，−3 d、−2 d）静脉滴注。

3. 移植物抗宿主病（GVHD）分级和防治：在rATG总量10 mg/kg的基础上，采用环孢素A或他克莫司联合短程甲氨蝶呤MTX为基础的GVHD预防方案，根据药物浓度监测、移植后嵌合度及原发病残留及复发情况动态调整免疫抑制剂用量。未发生GVHD的患者，移植后60 d开始环孢素A或他克莫司逐渐减量、停药。发生GVHD的患者，以甲泼尼龙1～2 mg·kg^−1^·d^−1^作为一线治疗首选用药，二线治疗包括CD25单抗、芦可替尼、西罗莫司等，酌情延长免疫抑制剂使用时间。急性GVHD及慢性GVHD分级参照文献[7]。

4. 移植后肿瘤残留、复发进展的处理：减停免疫抑制剂，并酌情予α干扰素诱导移植物抗肿瘤（GVT）效应。

5. 疗效评估标准：采用淋巴瘤PET-CT Deauville评分标准，Deauville评分为1、2、3分为CR；若氟代脱氧葡萄糖（FDG）代谢较基线降低但结果仍为4、5分，为PR，若治疗后代谢没有明显变化则被认为治疗无反应（NR），代谢升高或有新病灶出现被认为是疾病进展（PD）。中性粒细胞植入定义为连续3 d外周血中性粒细胞计数>0.5×10^9^/L。血小板植入定义为连续7 d血小板计数>20×10^9^/L且脱离血小板输注。

6. 随访及相关定义：通过查阅患者住院病历方式获得患者随访资料。末次随访时间为2022年12月1日。OS、DFS时间均从造血干细胞回输日开始计算。非复发死亡定义为由移植相关原因导致的与疾病进展/复发无关的死亡。

## 结果

一、一般资料

11例患者中男6例，女5例，中位年龄40（22～58）岁；外周T细胞淋巴瘤非特指型7例，血管免疫母细胞性T细胞淋巴瘤3例，蕈样真菌病（大细胞转化）1例；Lugano分期均为Ⅲ、Ⅳ期；8例患者有B症状；移植前均接受了多线化疗，中位治疗线程数为4（2～10）线，1例患者有自体造血干细胞移植史。移植前中位病程为17（6～36）个月。

二、造血重建情况

所有患者均成功植入。中位中性粒细胞植入时间为14.5（11～16）d，中位血小板植入时间为13（8～18）d。所有患者在移植后达到供者细胞完全嵌合。

三、疗效评估

11例患者移植前均为PD，移植后有9例（81.8％）最佳疗效达CR。1例移植后CR患者，移植后130 d原发病复发，予α干扰素治疗诱导GVT效应，后疾病评估持续CR。另2例患者分别于移植后36 d、77 d临床复发，移植后1年累积复发率（CIR）为（20.2±12.7）％。6例外周T细胞淋巴瘤非特指型患者移植后4例或得CR，1例未缓解（NR），1例死于多脏器功能衰竭，至随访终点5例存活。3例血管免疫母细胞性T细胞淋巴瘤患者移植后均获得CR，至随访终点全部存活。1例蕈样真菌病（大细胞转化）患者移植后36 d疾病复发，移植后101 d死亡。1例T大颗粒淋巴细胞白血病移植后获得CR，至随访终点存活。

四、生存情况

中位随访317（30～625）d。11例患者中，3例在移植后死亡，其中1例于移植后101 d死于疾病复发，另2例非复发死亡患者分别死于多脏器功能衰竭（移植后30 d）和移植相关血栓性微血管病（移植后117 d），移植后1年NRM为（22.5±14.0）％，OS率为（72.7±13.4）％，DFS率为（63.6±14.5）％。

五、预处理相关不良反应

所有患者预处理后均发生造血抑制。预处理相关不良反应均按药物毒副反应判定标准NCICTC2.0进行分级。非血液学不良反应最常见的是消化道反应：恶心呕吐（Ⅰ/Ⅱ级3例），黏膜炎（Ⅰ/Ⅱ级3例，Ⅲ/Ⅳ级1例），腹泻（Ⅰ/Ⅱ级2例，Ⅲ/Ⅳ级1例），有3例患者发生Ⅰ/Ⅱ级转氨酶/胆红素升高。感染是预处理后骨髓抑制期最常见的不良反应，7例患者在粒细胞缺乏期发生Ⅲ级感染：皮肤软组织感染1例，腹腔感染4例，尿路感染1例，肺部感染1例。上述非血液学不良反应经过治疗均得到缓解。

六、移植并发症

1. GVHD：11例患者中，移植后发生Ⅰ～Ⅱ度、Ⅲ/Ⅳ度急性GVHD各1例，予加强免疫抑制治疗后均获缓解。至随访终点，8例存活患者中4例发生慢性GVHD。

2. 巨细胞病毒（CMV）感染：移植后常规监测CMV-DNA，11例患者1年内CMV血症累积发生率为90.9％（10例），经更昔洛韦、磷甲酸等抗病毒治疗后CMV-DNA均转阴，未发生CMV病。

3. 其他移植并发症：1例患者移植后2个月出现移植相关血栓性微血管病（TA-TMA），1例患者于移植后1个月出现多脏器功能衰竭，2例患者均死亡。

## 讨论

ATG含有多种针对T细胞表面蛋白的抗体（包括CD2、CD3、CD4、CD5、CD7、CD8等），可以通过破坏再循环池中的T淋巴细胞发挥其免疫抑制作用，被广泛用于预防和治疗实质器官移植急性排斥反应，同时也被用于预防allo-HSCT急性GVHD。研究表明，ATG在体外能抑制T肿瘤细胞的增殖，并以剂量依赖性的方式诱导T肿瘤细胞的凋亡[8]–[9]，因此，预处理中足量使用ATG可能作为抗T细胞肿瘤的靶向治疗药物来克服化疗耐药问题，同时还能兼顾GVHD的预防。TBI是造血干细胞移植预处理一个重要手段，其目的在于通过非化疗手段杀灭恶性肿瘤细胞，同时清除患者骨髓造血功能、抑制免疫，为供者干细胞植入提供条件。TBI能在一定程度上克服化疗耐药，还可以在化疗药物不容易到达的区域（脑、睾丸等）达到治疗效果。本团队曾采用TBI/rATG/Vp16/CTX预处理方案allo-HSCT治疗23例高侵袭性T淋巴系统肿瘤（包括16例T淋巴母细胞淋巴瘤/急性淋巴细胞白血病和7例PTCL），移植后CR率为95.7％，中位随访25个月中，16例（69.6％）患者无病存活[10]。但该研究的研究对象大部分为T淋巴母细胞淋巴瘤/急性淋巴细胞白血病，疾病状态包括CR、PR及NR。本研究结果显示，使用TBI+rATG为基础的联合预处理方案haplo-HSCT治疗耐药进展期PTCL，供者造血细胞均顺利植入，预处理相关不良反应发生率也不高于其他清髓性预处理方案[11]，未出现非预期的不良反应。移植后1年NRM为（22.5±14.0）％，提示该预处理方案具有良好的安全性。通常rATG在用于haplo-HSCT患者GVHD预防时的总剂量为6～7.5 mg/kg，但为了尽量使rATG的抗肿瘤作用发挥到最大，我们采用总剂量10 mg/kg rATG并联用TBI为基础的预处理方案，以期协同发挥非化疗抗肿瘤作用、克服肿瘤耐药性。1例患者移植后复发，经过诱导GVT作用重新获得缓解，也证实了GVT对PTCL有较好作用。本研究中，移植后2个月即开始减停免疫抑制剂，目的是为了使GVT效应提供持久的疾病控制。

本组病例移植后CMV激活率较高，考虑与较大剂量rATG使用导致的免疫缺陷有关，但经过临床抢先治疗，未出现危及生命的病毒感染并发症。移植后急性GVHD的发生率低于近期同类研究[12]，但存活患者慢性GVHD发生率偏高，可能与免疫抑制剂早期减停有关。

本研究结果显示，TBI+rATG为基础预处理haplo-HSCT治疗化疗耐药进展期PTCL有效且安全性良好。本研究为小样本回顾性研究且缺乏对照，随访时间较短，上述结论尚需进一步确认验证。
